# Germline BRCA2 Mutation and Lynch Syndrome in a Patient With Multiple Primary Malignancies

**DOI:** 10.1155/crom/5879510

**Published:** 2025-08-20

**Authors:** Morgan M. Puglisi, Natasha N. Dziarnowski, Bohdan Baralo, Hetal Vachhani

**Affiliations:** ^1^Virginia Commonwealth University School of Medicine, Richmond, Virginia, USA; ^2^Department of Internal Medicine, Virginia Commonwealth University, Richmond, Virginia, USA; ^3^Division of Hematology, Oncology and Palliative Care, Virginia Commonwealth University Massey Comprehensive Cancer Center, Richmond, Virginia, USA

## Abstract

**Introduction:** Deficiency in *BRCA* genes leads to impediment in DNA repair and is associated with an increased lifetime risk of breast, ovarian, prostate, and pancreatic cancer and melanoma. Lynch syndrome is caused by inherited mutations in genes responsible for DNA mismatch repair, with resultant increase in lifetime risk of colorectal, endometrial, ovarian, stomach, urinary, pancreatic, and CNS malignancies. Here, we present a patient with a rare coexistence of both *BRCA2* and *PSM2* mutation in the setting of metastatic pancreatic and prostate cancer.

**Case Presentation:** The patient was initially diagnosed with metastatic prostate adenocarcinoma at the age of 57, when a screening PSA of 22.9 warranted biopsy and staging scans revealed involvement of the aortocaval lymph node. Treatment with androgen deprivation therapy with the addition of abiraterone and prednisone was initiated. Additionally, a PET scan showed a hypermetabolic isolated lung nodule, which upon wedge resection showed Stage I lung cancer. Three years later, a pancreatic mass and multiple liver lesions were found on a surveillance scan. Biopsy confirmed the diagnosis of pancreatic cancer, and germline testing revealed the coexistence of *BRCA2* and *PMS2* mutations. The patient completed four cycles of cisplatin and gemcitabine, followed by the initiation of olaparib as per the POLE study. About 12 months after the diagnosis of metastatic pancreatic cancer and 4 years after the diagnosis of metastatic prostate cancer, the patient has excellent control of the disease with performance status ECOG 0 and minimal side effects from maintenance therapy.

**Conclusion:** This case presents a unique combination of two coexistent inherited syndromes with the approximate combined incidence of 1:357,000. The presence of two advanced malignancies in our patient underlines the cumulative effects of combined deficiency in the DNA repair pathway and possibly an even higher lifetime risk of cancer. This may explain the presence of lung cancer with a minimal smoking history of less than 10 pack years. PARP inhibitors may be effective in controlling metastatic pancreatic cancer, and thus, genetic counseling is important for this patient and his family members, who will need appropriate cancer screening. The probability of carrying two pathogenic variants may be expected to increase as a result of next-generation sequencing and germline testing.

## 1. Introduction

Deficiency in *BRCA* genes leads to impediment in DNA repair and is associated with an increased lifetime risk of breast, ovarian, prostate, and pancreatic cancer and melanoma [1]. Lynch syndrome is caused by inherited mutations in genes responsible for DNA mismatch repair (MMR), which cause an increased lifetime risk of colorectal, endometrial, ovarian, gastric, urinary, hepatobiliary, pancreatic, and CNS malignancies [2, 3]. The estimated incidence of Lynch syndrome in the Western population is 1:370–1:2000 [4]. For germline mutations in *BRCA1* and *BRCA2*, the reported incidence is 1:400–1:800 [5].

## 2. Case Description

Here, we present a patient with a rare coexistence of both germline *BRCA2* and *PMS2* mutations in the setting of three primary malignancies (prostate, lung, and pancreatic); two of which were metastatic at the time of presentation (prostate and pancreatic).

The patient is an African American male with a past medical history of alcohol use disorder and nicotine dependence, with a smoking history of less than 10 years. He also gave a history of asbestos exposure on construction and pulmonary nodules. When he presented at the age of 58, he was found to have a PSA of 17 on a screening test. Family oncology history was positive for breast cancer in his mother and sister and prostate cancer in both his brothers. Patient was not aware, if any of his family member had comprehensive genetic evaluation. Screening performed 1 year earlier showed two rectal tubulovillous adenomas.

MRI of the prostate showed Prostate Imaging Reporting and Data System (PI-RADS) 4 lesions in the right posterior peripheral zone, as well as left pelvic adenopathy and lymph node enlargement at the iliac bifurcation. Biopsy confirmed diagnosis of prostate adenocarcinoma Grade 2 (Gleason 3 + 4). The patient was referred to radiation oncology for curative treatment. However, the Axium PET-CT scan performed before the radiation showed involvement of the aortocaval lymph node. Given this finding, the patient was upstaged to Stage 4 metastatic cancer, so systemic treatment with androgen deprivation therapy (leuprolide) and abiraterone acetate with prednisone was initiated.

Eight months later, the patient was admitted for upper gastrointestinal bleeding, and CT scans performed on admission showed an increase in size of the left upper lobe nodule (14 × 15 mm). The follow-up PET-CT showed increased activity (SUV max 3.22), and biopsy confirmed the diagnosis of bronchogenic adenocarcinoma. Wedge resection of the left upper lobe was performed approximately 1 year after the initial diagnosis of lung cancer. The final pathology showed Stage 1 adenocarcinoma with visceral pleura involvement.

Thirty months after prostate cancer diagnosis, the patient presented to the emergency department for hematochezia. The abdominal imaging revealed concern for a cystic pancreatic mass and multiple liver lesions. The liver lesion biopsy revealed adenocarcinoma consistent with pancreaticobiliary differentiation, followed by a pancreatic mass biopsy confirming the diagnosis of pancreatic adenocarcinoma. The patient was started on systemic treatment with nab-paclitaxel and gemcitabine. The commercially available germline testing based on next-generation sequencing revealed *BRCA2* (*c.4127-4130del* (*p.Gly1376ALAfs*∗*11*)) and *PMS2* (*deletion in Exons 6-8*) mutations. The point of care testing was requested by the treating oncologist with comprehensive geneticist evaluation after result was available. Treatment was switched to cisplatin and gemcitabine for 4 months and later to olaparib maintenance as per POLE study.

The follow-up scans were consistent with stable disease 10 months after initiating olaparib maintenance and ongoing abiraterone treatment with no evidence of new nodules in the chest, undetectable PSA, and no dose-limiting toxicities.

## 3. Discussion

Lynch syndrome typically occurs due to mutations in the DNA MMR genes, including *MLH1*, *MSH2*, *MSH6*, and *PMS2* [6]. This leads to an increased risk of multiple types of cancer, including colorectal, pancreatobiliary, endometrial, ovarian, stomach, and small bowel [7]. The data on the association of prostate cancer in patients with Lynch syndrome is mixed, suggesting that patients with the *MSH2* mutation can have an increased risk for prostate cancer, whereas patients with other mutations have a similar risk to the general population [8, 9]. Cases of lung cancer in patients with Lynch syndrome have been reported, but these appear to be spontaneous [10, 11]. Despite the increased risk for cancer, data suggest patients with Lynch syndrome who develop colorectal cancer actually have a better long-term survival prognosis compared to patients who develop sporadic colorectal cancer, which could be precipitated by increased surveillance for patients with Lynch syndrome [12]. However, among all MMR deficiencies, *PMS2* mutation tends to be more aggressive in pathogenesis, leading to worse prognosis than colorectal cancers with other MMR mutations [13].

The relative risk of cancer depends on the specific pathogenic mutation and location of primary tumors. While some mutations do not pose any risk at all, as seen in the cases of *MSH2*, *MSH6*, and *PMS2* in the development of pancreatic cancer, the *MLH1* mutation carries a 3.65 times higher chance of developing pancreatic cancer. The summary of relative malignancy risks in carriers of different Lynch syndrome mutations, as well as *BRCA1* and *BRCA2* carriers, can be found in Table 1 [14–21].

Current guidelines suggest universal testing of all new colorectal cancer diagnoses for mismatch repair deficiency (dMMR) [2]. If screening tests of DNA MSI or MMR protein IHC are positive, germline DNA testing is completed to determine a Lynch syndrome diagnosis [3]. Testing of family members is indicated given the implication of early screening in carriers.

For individuals who are *MLH1* or *MSH2* mutation carriers, colonoscopy is recommended every 2 years starting at age 25 or age 35 if they are an *MSH6* or *PMS2* variant carrier [2]. In addition, consideration of daily aspirin is recommended to reduce the long-term risk of colorectal cancer [22]. Due to the high risk of endometrial cancer, hysterectomy and bilateral salpingo-oophorectomy can be considered for women aged 40 years who are *MLH1*, *MSH2*, and *MSH6* variant carriers and for women aged 50 years who are *PMS2* carriers [22].

Treatment with a combination of anti-PD-1 and anti-CTLA4 agents (ipilimumab-nivolumab) or anti-PD-1 agents (nivolumab, pembrolizumab, and dostarlimab) is suggested in the first line for all patients with MMR-deficient metastatic colorectal cancer [23–25]. Anti-PD-1 agents are also used for neoadjuvant treatment of locally advanced rectal cancer for downstaging purposes. In the instance of a complete response in patients with colorectal cancer, surveillance can be pursued [26, 27]. Dostarlimab, in the recent Phase 2 clinical trial among patients with advanced rectal cancer, showed a complete response in all 41 patients who completed the treatment [28].

The use of PD-1 inhibitors (dostarlimab and pembrolizumab) in conjunction with chemotherapy is now utilized in patients with MMR-deficient advanced and recurrent endometrial cancer [29, 30].

Many patients with MMR-deficient advanced or metastatic cancer treated in the past with chemotherapy can be candidates for treatment with single-agent anti-PD-1 therapy in tumor-agnostic way [31–34].

The *BRCA1* and *BRCA2* mutations have been associated with hereditary breast and ovarian cancer as well as earlier onset of these malignancies [1, 35]. About 66% of patients have the *BRCA1* gene and 34% possess the *BRCA2* gene variant [35]. However, the *BRCA2* mutation in particular has also been implicated in increasing the risk of pancreatic, prostate, gastric, laryngeal, fallopian tube, and malignant skin cancers as well as eye melanoma [1]. Data estimates that patients with the *BRCA2* mutation have a threefold risk of prostate cancer compared with the general population and tend to have more aggressive prostate cancers [36, 37]. It is estimated that patients with the *BRCA2* mutation have a 7.2-fold risk of pancreatic cancer compared to the general population [38].

Early detection of *BRCA* mutations can also be helpful in taking measures to reduce cancer risk and increase surveillance to limit cancer burden on patients [35]. Current guidelines suggest multiple factors that should raise suspicion for *BRCA1* and *BRCA2* variants:

• Breast cancer diagnosis at age ≤ 50 years old
• Multiple primary breast cancers
• Tripe-negative breast cancer
• Male breast cancer
• Any ovarian cancer
• Breast cancer in individual of Ashkenazi Jewish ancestry
• Combination of prostate cancer and/or prostate cancer (Gleason score ≥ 7 or metastatic) with breast cancer and/or ovarian cancer
• 2+ relatives with breast cancer with one diagnosed at age ≤ 50 years old
• 3+ relatives with breast cancer
• Family history of *BRCA1* or *BRCA2* variant

*BRCA1* and *BRCA2* gene variants can be detected with the use of a gene panel involving sequence analysis and deletion/duplication analysis [35]. If a patient possesses a *BRCA1* or *BRCA2* variant, monthly breast self-examinations, clinical breast examination, annual mammography, and breast MRI are recommended to screen for breast cancer in women, starting at age 30 or 5 years prior to the incidence of cancer in the youngest relative [39]. For *BRCA1* carriers, 6-month screening with MRI should be considered [39]. Male carriers should be offered annual blood PSA screening starting at the age of 40 years, and they should be encouraged to recognize and report any physical breast changes. Screening for pancreatic cancer can be considered for patients who have a first- or second-degree relative with exocrine pancreatic cancer, beginning at age 50 [39]. For the prevention of cancers in women with *BRCA* variants, ESMO clinical practice guidelines indicate that prophylactic bilateral mastectomy should be considered, and risk-reducing bilateral salpingo-oophorectomy is recommended for women with *BRCA1* variants between the ages of 35 and 40 and with *BRCA2* variants between the ages of 40 and 45. In addition to consideration of risk-reducing mastectomy and salpingo-oophorectomy, USPSTF guidelines recommend offering female patients with *BRCA* variants risk-reducing medications, including tamoxifen, raloxifene, or aromatase inhibitors [40].

For women with *BRCA* variants who present with Stage I–III high-risk breast cancer, breast radiation or risk-reducing mastectomy should be considered [39]. The introduction of the poly ADP ribose polymerase (PARP inhibitors) opens new treatment options for patient with germline or somatic *BRCA* mutation in breast, prostate, pancreatic, and ovarian cancers, many of which are FDA approved.

For germline *BRCA*-mutated, HER-2-negative breast cancer, two PARP inhibitors are currently approved. First, talazoparib is approved as a single-agent therapy for locally advanced or metastatic breast cancer [41]. Second, olaparib is approved as a maintenance regimen for patients who have been treated with chemotherapy in neoadjuvant, adjuvant, or metastatic settings [42, 43].

In patients with somatic and germline *BRCA*-mutated castrate-resistant prostate cancer, olaparib and rucaparib are currently approved by the FDA [44, 45].

In patients with germline *BRCA*-mutated metastatic pancreatic cancer, as in our case, olaparib was approved as a maintenance regimen after at least 16 weeks of a first-line cisplatin-based regimen [46].

Olaparib was approved as maintenance in patients with germline and somatic *BRCA*-mutated advanced epithelial ovarian, fallopian tube, and primary peritoneal cancer who had responded to the first-line platinum-based chemotherapy [47]. Lastly, olaparib is indicated in the third and beyond line systemic treatment of advanced epithelial ovarian, fallopian tube, and primary peritoneal cancer, as a monotherapy [48].

Coexistence of Lynch syndrome and *BRCA* mutations is uncommon, with only one other report available in the literature [49]. It has been found that the coexistence of *BRCA* mutations in patients with *MLH1*, *MSH2*, and *MSH6* variants increases the risk for extracolorectal cancers [50]. A female patient with *BRCA2/PMS2* mutations developed both breast cancer and colorectal cancer [49]. Given the scarce information on cancer risk in patients with concurrent *BRCA2* and *PMS2* mutations, it is hard to state whether lung cancer diagnosis in the patient is related to the germline mutations or environmental exposures (tobacco, asbestos).

## 4. Conclusion

The combination of Lynch syndrome and *BRCA* germline variants is rare but can be present in patients with multiple foci of cancer, as seen in our case. The cumulative effects of combined deficiency in the DNA repair pathway create an even higher lifetime risk of cancer. This may explain the presence of lung cancer with a minimal smoking history of less than 10 pack years and brief asbestos exposure in the youth. PARP inhibitors are likely to be effective in controlling metastatic pancreatic cancer. Genetic counseling is important for this patient and his family members, who will need appropriate cancer screening. The probability of carrying two pathogenic variants may be expected to increase as a result of next-generation sequencing tests.

## Figures and Tables

**Table 1:** Relative risk of malignancies based on the BRCA mutation and mismatch repair deficiency subtype.

	**Incidence in population**	**RR of prostate cancer**	**RR of pancreatic cancer**	**RR of colon cancer**	**RR of endometrial cancer**	**RR of gastric cancer**	**RR of ovarian cancer**	**RR of urothelial cancer**	**RR of small intestine cancer**	**RR of brain tumors**	**RR of breast cancer**
BRCA1	0.20% 1:500 [14]	< 65 years: 1.82 [15]	2.17 [16]	1.48 [16]	—	2.17 [16]	10.55 [17]	—	—	—	Female breast: 8.39–47.45 [18] Male breast: 4.30 [16]
BRCA2	0.45% 1:222 [14]	2.22 [16]	3.34 [16]	—	—	3.69 [16]	2.57 [17, 18]	—	—	—	Female breast: 2.66–31.85 [18] Male breast: 44.0 [16]
MLH1	0.05% 1:1946 [19]	0.35–1.10 [20]	3.65 [20]	11.2–14.9 [20]	11.0–17.4 [20]	6.25–8.75 [20]	3.64–18.2 [20]	0.87–3.04 [20]	1.33–36.7 [20]	1.4–3.4 [20]	—
MSH2	0.04% 1:2841 [19]	0.31–1.89 [20]	0.29–0.94 [20]	8.05–12.7 [20]	6.77–18.4 [20]	0.25–11.3 [20]	6.77–18.4 [20]	1.91–5.57 [20]	3.67–33.3 [20]	5–15.4 [20]	—
MSH6	0.13% 1:758 [19]	0.20–0.92 [20]	0.82–0.94 [20]	2.44–10.7 [20]	5.16–15.8 [20]	1.25–9.88 [20]	0.91–11.8 [20]	0.43–3.57 [20]	3.33–13.3 [20]	1.6–3.6 [20]	—
PMS2	0.14% 1:714 [19]	0.37–0.92 [20]	0.59–0.94 [20]	2.12–4.88 [20]	4.19–8.39 [20]	—	2.12–4.88 [20]	0.43–1.04 [20]	0.33–1.00 [20]	1.2–2 [20]	—

*Note:* Relative risks for cancers associated with Lynch syndrome mutations calculated from cumulative incidence in patients with mutations and the general population. Relative risks for ovarian cancer associated with BRCA1 and BRCA2 calculated from odds ratio and nonexposed prevalence.

## Data Availability

Data sharing is not applicable to this article as no new data were created or analyzed in this study.
